# A Case of Traumatic Tetanus Successfully Treated with Calabar Bean

**Published:** 1873-11

**Authors:** J. A. Duncan

**Affiliations:** Toledo, O.


*A Case of Traumatic Tetanus Successfully Treated with Calabar Bean.* By J. A. Duncan, M.D., Toledo, O. (Read before the Toledo Medical Society.)

Mr. J. A., aged 17, German laborer. On June 13th, 1873, became faint (from ague) and fell with his right fore-arm against a circular saw, which nearly severed it just above the wrist. Amputation was made an inch above the injury soon after. Late in the afternoon of same day, having symptoms of ague, I gave him ordinary doses of quiniæ sulph. and iron, and they did not return. The wound seemed to do well, and on the fourth day he walked half a mile to my office to get it dressed. He continued to do so until the thirteenth day from injury; this day he complained of pain in the right ear. As his tongue was coated, and he had taken no quinine for eight days, I suspected a relapse of ague, and gave him 5 gr. doses of quinine every three hours. The pain in the ear ceased at the third dose.

At 9 A. M. the next day (fourteenth from injury), on reaching for his hat to come to my office he was seized with a spasm and fell to the floor. I arrived in fifteen minutes, and found him suffering from most intense pain in epigastrium, at base of brain, and down the entire spine. The body was bent backwards, in a state of rigid opisthotonos. Could open his jaws some, but trismus was complete in an hour.

The brows were corrugated, and the "risus sardonicus" was well marked. Respiration was so difficult, from the muscular spasms, that I thought at times he would suffocate. During the four following days so severe were the paroxysms that he would be thrown violently to one side, nor was the tonic spasm of the muscular system completely relaxed at any time.

Gave first a cathartic of podophyllin and rhubarb, and followed with enormous doses of opium, hydrate of chloral, belladonna, ether and bromide of potassium, during these four days, without apparent benefit. It was then advised to resort to the calabar bean. A tincture was obtained, containing one grain of the powdered bean to m. iv of the menstruum. Gave 14 drops at once. Retention of urine for twenty-six hours seemed to require attention, but I was unable to pass the catheter, on account of the spasmodic contraction at neck of bladder. Tincture of calabar bean was ordered in 9 drop doses every two hours. After the lapse of four hours I found patient almost relaxed, and that he had passed more than a quart of urine, voluntarily, about half an hour after the first dose. The pulse had fallen from 104 to 80, and there was not half the pain complained of previously.

Continued treatment. Next day found he had slept two or three hours during the night. Added quinine grs. iii, + tr. ferri chlor., m. vi., alternated with tr. of calabar bean. Sixth day (of tetanus) no better; ordered 12 drops of the latter every two hours. Seventh day, found him asleep, with pulse at 80; had now slept seven consecutive hours, and had taken no medicine for that length of time. He awoke with a spasm, and his pulse rose in one minute to 140! As the succeeding spasms were worse than on previous day, I gave 15 drops of tr. calabar bean at once; in half an hour he was better; continued the same dose every two hours. Called at 4 P. M. same day: found his eyes glaring; respiration 16, pulse 54, and free from pain, but with occasional slight twitchings of limbs.

Reduced dose to ten drops every two hours, and continued it for three days. On ninth day of tetanus he refused to take medicine from 9 P. M. to 9 A. M. next day, when I found him much worse; pulse 120, respiration 35, sleepless, with frequent and intense spasms, constantly getting more so. Gave 15 drops of calabar bean + 15 grs. of hydrate of chloral, which soon relaxed the muscles, and he fell asleep. I ordered him to be awakened every two hours, and the dose (of c. b.) repeated. This was done for the three days following, from which time he improved rapidly, the chloral being given every four hours the first day, and only at night after the third day.

Twelfth day, much better. Gave tr. of calabar bean every three hours in 8 drop doses, and continued it for the next six days, without much change for the better. Nineteenth day, gave 15 drops every three hours for the entire day, and then discontinued it altogether, as this seemed to be the *coup de grace;* for, on the following day (twentieth day of tetanus) I found my patient sitting on the edge of the bed, just finishing a gallon bowl full of beef soup and sweet milk, with bread soaked in it, and "yet he was not happy!" He wanted more of the same sort, or else some *beer*, he didn't care which. Twenty-first day, able to walk around the yard, but was very weak and emaciated. Most of the time during the attack his body was covered with profuse and clammy perspiration, which was cool on the exposed surfaces of the body. I regret to say the temperature was not noted. Some stimulants and the usual nourishing liquid food were given through a gum catheter by the mouth, it being difficult to get it between his teeth at all times.

After the calabar bean was given he had no more urinary trouble. His bowels became regular after opium was discontinued. His appetite was good, and at times ravenous; *e. g.*, the seventh day of tetanus he took three quarts of boiled milk, immediately followed with one and a half pints of strong beef tea and two ounces of sherry wine; an hour afterwards two-thirds of a quart of dried cherry soup or tea. Prior to taking calabar bean, he experienced great difficulty in swallowing, but while under its influence could do so with comparative ease. On tenth day could open jaws one-fourth of an inch; twelfth day, could protrude tongue; at close of tenth day profuse sweating ceased. The wound had healed, except a small orifice at the centre as large as a goose quill, from which a little pus could be squeezed, at the end of third week from injury. On fourth day a little erysipelatous redness appeared on the fore-arm, and spread up the arm some, but at the end of seventh day from injury this had all gone, nor did it re-appear.

At no time was there any tenderness over the median, or other nervous trunks leading from the injured parts. There was tenderness over the unhealed portion of the stump at times, apparently over the anterior inter-osseous nerve.

Lastly, the injured arm or limb was never noticed to be affected with spasm. Evidently, from this and other reported cases, we have in the calabar bean a remedy which is a powerful and safe *spinal* and *arterial* sedative, which, if properly administered and combined with other agents, will save many cases of tetanus which would otherwise die.

---

Cold Infusion of Green Coffee in Gout.—Dr. Monchaux communicates to *L'Abeille Medicale* his experience in the use of cold infusion of green coffee in the treatment of gout. He directs his patients to take a tablespoonful of green coffee, place it in a tumbler, half fill the latter with water at the temperature of the atmosphere, and allow it to stand for twenty-four hours. In the morning the liquid is to be swallowed, an equal quantity of water added, and taken the following morning; thus the same quantity of coffee will serve for two doses. The liquid thus obtained has little savor; it is more or less distinctly green according to the species of coffee employed. Dr. Monchaux does not explain the mode of action of the remedy upon the uric diathesis. He inclines to the belief that it acts against the effects of the disease rather than on the disease itself, and that, in order to prevent a recurrence of gout, it is only necessary to make a daily use of the remedy indicated by him. He observes that the remedy is an old one, and that, however difficult it may be to explain the precise action, the efficiency of the remedy is acknowledged. He had himself used it during three years, and with the result that his bi-annual attacks of gout had completely disappeared.—*The Doctor.*

